# Sexual abstinence and associated factors among Myanmar youths: evidence from the Myanmar demographic and health survey 2015–16

**DOI:** 10.3389/frph.2026.1751178

**Published:** 2026-04-01

**Authors:** Yoon Shwe Yee Hlaing, Pallop Siewchaisakul, Sineenart Chautrakarn

**Affiliations:** Faculty of Public Health, Chiang Mai University, Chiang Mai, Thailand

**Keywords:** factors, Myanmar demographic and health survey, sexual abstinence, sexual and reproductive health, youths

## Abstract

**Objectives:**

Myanmar's youth's sexual behavior is a strong predictor of public health outcomes, particularly sexually transmitted infections, HIV/AIDS, and unintended pregnancies. Although abstaining from sex is a widely promoted preventive measure, there is little evidence among Myanmar's young people. This study sought to determine the prevalence of sexual abstinence and its associated factors.

**Methods:**

This retrospective study analyzed data from the 2015–2016 Myanmar Demographic and Health Survey (MDHS) to determine the prevalence of sexual abstinence and associated factors. The sample included 4,573 young people aged 15–24. Descriptive statistics, Univariable and multivariable logistic regression were used, with a significance of *p*-value < 0.05.

**Results:**

The findings indicated that 71.2% of Myanmar youths were sexually abstinent. Multiple logistic regression revealed that age 15–19 years (AOR = 0.13), females (AOR = 0.69), current tobacco use (AOR = 0.54), and moderate to high HIV/AIDS awareness level (AOR = 0.75, 0.73) were associated with reduced probabilities of sexual abstinence. Higher education level (AOR = 2.24), employment (AOR = 1.55), higher wealth index (AORs = 1.59–2.73), and access to media (AOR = 1.24) were associated with increased probabilities of sexual abstinence.

**Conclusion:**

These findings suggest the need for interventions that support school retention, address socioeconomic disadvantage, promote positive media engagement, and integrate substance-use prevention within sexual and reproductive health initiatives aimed at youths in Myanmar.

## Introduction

Youth sexual behavior is a significant determinant of public health outcomes, influencing not only individual well-being but also broader societal and developmental goals (1).‏ Globally, sexual activity among adolescents and young people (typically between 15 and 24 years old) has drawn increasing attention due to its association with sexual and reproductive health (SRH) risks, including HIV/AIDS, sexually transmitted infections (STIs), and unintended pregnancies (2).‏ Among various behavioral interventions, sexual abstinence, defined as refraining from voluntary sexual intercourse (3), is regarded as one of several strategies to reduce such risks (4).‏

Globally, the prevalence and patterns of sexual abstinence among youth vary greatly, influenced by cultural, religious, and sociodemographic factors (5).‏ Its importance is overemphasized in low- and middle-income countries, the places where resources for sexual health education and clinical care delivery may be scarce and where sociocultural norms and religious beliefs may further promote abstinence (6–8).‏ Well-organized, skill-oriented programs are anticipated to promote abstinence, facilitate youths in delaying their initial sexual experience, and overall decrease both the occurrence of sexual activities and the number of sexual partners (9).‏ The significance of sexual abstinence among youth is profoundly acknowledged in Southeast Asia, including Myanmar, where sociocultural paradigms, religious doctrines, and legal structures frequently underscore the necessity of postponing sexual initiation (10–12).‏

In Myanmar, around 9 million individuals fall within the 15–24 age group (youth), making up 18% of the population (13).‏ This demographic offers a myriad of opportunities as well as obstacles for the nation's health and development outlook. In 2022, an estimated 5,800 new HIV infections occurred among youths aged 15–24 years in Myanmar (14). According to World Bank data, the HIV prevalence among males and females aged 15–24 years was 0.3% in 2021 (15). Although nationally representative data on sexually transmitted infections (STIs) among youths are limited, STIs remain a recognized public health concern in Myanmar. These figures indicate a continued burden of HIV and related sexual health risks among Myanmar youths and highlight the importance of preventive behavioral strategies. Moreover, young people encounter difficulties in obtaining information and services related to sexual and reproductive health (SRH). These challenges are compounded by social and gender norms, which hinder young people, especially girls and other vulnerable groups, from attaining good health and well-being (16).‏

Globally, there were many studies about factors associated with sexual abstinence among youths. Studies indicated the association of sexual abstinence with sociodemographic profiles [e.g., age, sex, residence (urban/rural), education level, mass media exposure, etc.] (6, 17, 18).‏ Previous research showed that sexual abstinence was linked with knowledge of HIV/ AIDS and STIs (6, 17, 19).‏ These researches gave insights into youth's sexual abstinence and its associated factors and provided sexual and reproductive health measures in their respective countries.

There were some studies related to youth sexual health in Myanmar. One study focused on youths in Yangon, investigating factors related to the intention to prevent risky sexual behaviors (20).‏ Similarly, another study conducted a study focusing on underprivileged youth in Mandalay City (21).‏ Also, another study investigated the preference for abstinence-only or comprehensive sex education at public high schools in Pyin Oo Lwin Township (12).‏ However, these studies utilized small sample sizes that represented specific townships, so their findings don't represent the entire country. Moreover, there is a lack of study or research regarding sexual abstinence among youths in Myanmar. Therefore, there is a need for more comprehensive nationwide research on sexual abstinence among youths.

In sociocultural environments where premarital sexual activity is highly stigmatized and abstinence is promoted through religious, cultural, and legal standards, abstinence remains the prevalent behavioral expectation among youths (6, 10). However, research from other low- and middle-income nations suggests that abstinence habits are heavily influenced by education, socioeconomic status, media exposure, and health information, and that they may shift as societies go through social and economic transitions (6, 17). Without nationally representative evidence, SRH policies and initiatives may rely on assumptions rather than empirical data. Population-level research on sexual abstinence and its associated determinants is thus required to guide culturally relevant, evidence-based SRH measures that support the maintenance of protective behaviors while also addressing rising sexual health risks among Myanmar youths.

As a result, this study relied on data from the Myanmar Demographic and Health Survey (MDHS) 2015–2016, a statewide survey that includes a variety of variables relating to sexual and reproductive health, as well as HIV/AIDS. The MDHS was carried out in 2015–16, with the full report released in 2017 (22).‏ This study aimed to identify the prevalence of sexual abstinence among Myanmar teenagers. Furthermore, the study sought to establish a link between sexual abstinence and factors such as sociodemographic profiles, HIV/AIDS knowledge, STI awareness, and contraception knowledge among Myanmar's youth. The findings of this study can assist health officials in developing and implementing public policies focused on enhancing reproductive and sexual health education and services for Myanmar's youth.

## Methods

### Study design and setting

This retrospective analysis made use of data from the 2015–16 Myanmar Demographic and Health Survey (MDHS), which is nationally representative. The MDHS was carried out from December 7, 2015, to July 7, 2016, and the entire report was released in 2017. Furthermore, the MDHS was the first and most recent assessment of Myanmar's demographic health. The dataset included Naypyidaw Union Territory, as well as seven Myanmar states and provinces (22). This study used MDHS data to investigate sexual abstinence and related factors among Myanmar youths aged 15–24.

### Data source, study population, and sampling

The MDHS employed a two-stage stratified cluster sampling approach. In the first stage, clusters/enumeration areas (EAs) were chosen using equal probability systematic sampling with independent selection within each stratum. In the second stage, a predetermined number of households were chosen systematically from each cluster. For 2015–16, 442 clusters (123 urban and 319 rural) were selected (22). This study used the Individual Recode files for women (IR), men (MR), and households (PR). All of the data sets were obtained from the DHS program website (https://www.dhsprogram.com/).

In the original MDHS, 17,622 participants aged 15–49 were chosen at random from each household. The current study's population consisted of Myanmar youths aged 15–24. Male and female youths aged 15–24 years old with complete sociodemographic profiles, HIV/AIDS and STI awareness, contraceptive knowledge, and sexual abstinence were eligible. Individuals aged 15–24 with missing data on critical study factors were eliminated. As a result, this study comprised 5,186 participants aged 15–24 out of a total of 17,622. After deleting records with missing values of 541 participants, the unweighted sample size was 4,645 individuals. The survey weights provided by DHS were utilized to adjust for the complex sample methodology and assure nationally representative results. After applying survey weights, the final weighted sample consisted of 4,573 people aged 15–24.

### Study variables

In this study, we defined the outcome variable “sexual abstinence” as primary abstinence and classified it as No (had sex) or Yes (did not have sex). In MDHS, youths were asked, “How old were you when you had your first sexual experience?” The responses were coded as 0 (not having sex) and numbers (age of first sexual intercourse).

The independent variables were sociodemographic profiles [age group, sex, region, residence (urban/rural), education level, employment status, wealth index, media exposure, current tobacco use], HIV/AIDS knowledge and STI awareness, and contraception knowledge.

In the original database, the media exposure question was either No or Yes, with “Yes” indicating exposure to at least one of three media: watching television, listening to the radio, or reading a newspaper (23, 24).

HIV/AIDS knowledge was assessed using 11 variables covering knowledge of HIV transmission and prevention, including condom use, having one faithful uninfected partner, common misconceptions (e.g., mosquito bites, sharing food, supernatural causes), mother-to-child transmission (during pregnancy, delivery, and breastfeeding), and knowledge of HIV testing sites. Each “Correct” or “Yes” response was scored as 1 and each “Not correct” or “No” response as 0, yielding a composite score ranging from 0 to 11. Level of knowledge of HIV/AIDS was categorized into three levels according to Bloom's cut-off criteria (25): low (0–6; <60%), moderate (7–8; 60%–79%), and high (9–11; 80%–100%). STI awareness was measured using a single variable assessing whether respondents had ever heard of sexually transmitted infections other than HIV/AIDS (Yes/No).

Contraception knowledge was assessed using four variables: knowledge of any contraceptive method, and exposure to family planning messages through radio, television, and newspapers or magazines. Each positive response was coded as 1 and negative responses as 0, resulting in a composite score ranging from 0 to 4. The overall level of contraception knowledge was classified according to Bloom's cut-off criteria (25) into three categories: low (0–1; <60%), moderate (2; 60%–79%), and high (3–4; 80%–100%).

### Statistical analysis

Data was processed and analyzed using STATA version 15.1. This study made use of Individual Recode datasets for women (IR) and men (MR), as well as Household Recode files (PR). The datasets were merged using their unique identifiers (cluster number, household number, and individual line number within households).

Following the most recent DHS guidelines (DHS-8), all data were weighted using the v005/1,000,000 and mv005/1,000,000 scaling factors, and the STATA survey commands (svyset) were used to account for the survey's complex sampling design. This method was used to properly adjust for clustering, resulting in accurate estimation of confidence intervals and *p*-values according to DHS guidelines (26).

Descriptive statistics of frequency and percentage were used to determine the prevalence of sexual abstinence, sociodemographic profiles, HIV/AIDS and STI knowledge, and contraception knowledge among Myanmar's youth. The chi-squared test was used to assess the relationship between independent variables such as sociodemographic profiles, HIV/AIDS and STIs knowledge, and contraception knowledge, and the outcome variable, sexual abstinence. Univariable logistic regression was conducted as an initial screening step to identify potential associations between independent variables and sexual abstinence, and the results were presented as crude odds ratios (COR) with 95% confidence intervals (CI). Multivariable logistic regression was subsequently applied to control for potential confounding and to estimate independent associations, which is appropriate for a dichotomous outcome variable, and the results were presented as adjusted odds ratios (AOR), with 95% CI. Statistical significance was determined at a *p*-value of <0.05.

## Results

### Sociodemographic

The distribution of the 4,573 weighted sample youths was nearly equal between the age groups of 15–19 (49.2%) and 20–24 (50.8%). The majority were females (72.1%). Two-thirds of youths (66.7%) came from rural areas, with a sizable proportion from the delta and lowland regions (45.7%). The majority of participants (62.9%) had completed secondary education and were employed (63.9%). The wealth index was fairly distributed: 14.9% were in the poorest category, 17.3% poorer, 22.3% middle, 22.6% richer, and 22.9% richest. Only 8.2% smoked cigarettes, 0.2% chewed tobacco, and 8.4% consumed tobacco products. In terms of media exposure, 22.4% of participants read newspapers or magazines, 30.5% listened to the radio, and 69.7% of young people watched television. Overall, 77.9% of young people were exposed to at least one form of media in the previous year (Table 1).

**Table 1:** Sociodemographic profiles (*n* = 4,573, weighted).

Variables	*n*	Percentage (%)
Age group		
15–19	2,251	49.2
20–24	2,322	50.8
Sex		
Male	1,276	27.9
Female	3,297	72.1
Region		
Delta and lowland	2,092	45.7
Hilly	615	13.5
Coastal	367	8.0
Plains	1,499	32.8
Residence (Urban/rural)		
Urban	1,523	33.3
Rural	3,050	66.7
Education Level		
Low Education Level	1,299	28.4
High Education Level	3,274	71.6
Employment Status		
Unemployment	1,653	36.1
Employment	2,920	63.9
Wealth index		
Poorest	680	14.9
Poorer	791	17.3
Middle	1,019	22.3
Richer	1,035	22.6
Richest	1,048	22.9
Media exposure		
No	1,012	22.1
Yes	3,561	77.9
Currently smoking cigarettes		
No	4,196	91.8
Yes	377	8.2
Currently chewing tobacco		
No	4,562	99.8
Yes	11	0.2
Currently using tobacco products		
No	4,189	91.6
Yes	384	8.4

### HIV/AIDS knowledge and STI awareness

Table 2 revealed that all young people had heard of HIV/AIDS. However, this analysis revealed common misconceptions about HIV transmission. Overall, 38.3% of respondents had high HIV/AIDS knowledge, 32.0% had moderate knowledge, and 29.7% had low knowledge. Only 20.4% of respondents reported they were aware of STIs other than HIV and AIDS.

**Table 2:** HIV/AIDS knowledge and STI awareness (*n* = 4,573, weighted).

Variables	*n*	Percentage (%)
Ever heard of HIV/ AIDS		
No		0.0
Yes	4,573	100.0
Reduce chances of getting HIV by using condom		
Not correct	1,610	35.2
Correct	2,963	64.8
Reduce chance of getting HIV by having just one uninfected sex partner		
Not correct	1,183	25.9
Correct	3,390	74.1
Can get HIV from mosquito bites		
Not correct	2,770	60.6
Correct	1,803	39.4
Can get HIV by sharing food		
Not correct	1,896	41.5
Correct	2,677	58.5
Can a healthy-looking person have HIV		
Not correct	1,591	34.8
Correct	2,982	65.2
HIV can be transmitted during pregnancy		
Not correct	1,222	26.7
Correct	3,351	73.3
HIV can be transmitted during delivery		
Not correct	1,865	40.8
Correct	2,708	59.2
HIV can be transmitted by breastfeeding		
Not correct	1,469	32.1
Correct	3,104	67.9
Know a place to be tested for HIV		
No	1,555	34.0
Yes	3,018	66.0
Can get HIV by witchcraft or supernatural means		
Not correct	1,069	23.4
Correct	3,504	76.6
Level of knowledge of HIV/AIDS		
Low (0–6)	1,357	29.7
Moderate (7–8)	1,465	32.0
High (9–11)	1,751	38.3
Awareness of other STIs		
No	3,641	79.6
Yes	932	20.4

### Contraception knowledge

Table 3 showed that the vast majority of young people (97.0%) are aware of at least one modern or traditional contraceptive method. Less than one-quarter had limited exposure to family planning messages through various media in the previous year. In terms of contraceptive knowledge, 16.4% of participants showed a high level, 19.9% had moderate knowledge, and 63.7% had a low level.

**Table 3:** Contraception knowledge (*n* = 4,573, weighted).

Variables	*n*	Percentage (%)
Knowledge of any contraceptives method		
Don't know any method	139	3.0
Know any method (Modern or Traditional)	4,434	97.0
Heard family planning (FP) on radio last months		
No	4,032	88.2
Yes	541	11.8
Heard FP on TV last months		
No	3,473	75.9
Yes	1,100	24.1
Heard FP on newspaper or magazine last months		
No	3,619	79.1
Yes	954	20.9
Level of knowledge of contraception		
Low (0–1)	2,914	63.7
Moderate (2)	910	19.9
High (3–4)	749	16.4

### Prevalence of sexual abstinence

Among the 4,573 weighted sample youths, 3,255 (71.2%) reported abstaining from sexual activity.

### Factors associated with sexual abstinence

Several significant associations were identified in both univariable and multivariable logistic regression analyses. Older youths are less likely to practice sexual abstinence, with an adjusted odds ratio (AOR) of 0.13 (95% CI: 0.11–0.16, *p* < 0.001) compared to those aged 15–19 years. Females were significantly less likely than males to report abstinence (AOR = 0.69, 95% CI: 0.56–0.86, *p* = 0.001). Youths with a high level of education were more likely to abstain than those with a low level of education (AOR = 2.24, 95% CI: 1.78–2.81, *p* < 0.001). Employment was a significant predictor of abstinence, with employed youths being more likely to abstain than unemployed youths (AOR = 1.55, 95% CI: 1.26–1.91; *p* < 0.001). Economic status, as measured by the wealth index, showed a significant positive association: compared to the poorest group, those in higher wealth quintiles had higher odds of abstaining. The AORs were 1.59 (95% CI: 1.16–2.17, *p* = 0.004) for the poorer group, 1.67 (95% CI: 1.21–2.30, *p* = 0.002) for the middle group, 1.46 (95% CI: 1.05–2.04, *p* = 0.026) for the richer group, and 2.73 (95% CI: 1.84–4.05, *p* < 0.001) for the richest. Youths with access to at least one form of media (newspapers, radio, or television) at least once per week had higher odds of abstinence (AOR = 1.24, 95% CI: 1.01–1.53; *p* = 0.043) than those with no access. Non-users of tobacco products were much more significantly likely to practice abstinence than their peers who currently used tobacco (AOR = 0.54, 95% CI: 0.37–0.77; *p* = 0.001).

Knowledge factors were also significant. Awareness of other STIs, apart from HIV, was associated with lower odds of abstinence (AOR = 0.78, 95% CI: 0.62–0.97, *p* = 0.028). In addition, youth with moderate (AOR = 0.75, 95% CI: 0.59–0.95, *p* = 0.016) and high (AOR = 0.73, 95% CI: 0.57–0.94, *p* = 0.013) levels of HIV/AIDS knowledge had lower odds of abstinence compared to those with low knowledge (Table 4).

**Table 4:** Factors associated with sexual abstinence.

Variables	Crude OR (95% CI)	*P*-value	Adjusted OR (95% CI)	*P*-value
Age group				
15–19	ref		ref	
20–24	0.14 (0.12–0.16)	<0.001^a^	0.13 (0.11–0.16)	<0.001^a^
Sex				
Male	ref		ref	
Female	0.77 (0.65–0.90)	0.001^a^	0.69 (0.56–0.86)	0.001^a^
Region				
Delta and lowland	ref		ref	
Hilly	0.82 (0.60–1.10)	0.188	0.77 (0.54–1.11)	0.158
Coastal	0.75 (0.58–0.97)	0.029^a^	1.07 (0.77–1.48)	0.689
Plains	1.08 (0.88–1.33)	0.469	1.05 (0.82–1.33)	0.714
Residence (urban/rural)				
Urban	ref		ref	
Rural	0.71 (0.58–0.86)	0.001^a^	1.11 (0.85–1.46)	0.453
Education level				
Low education level	ref		ref	
High education level	2.58 (2.16–3.09)	<0.001^a^	2.24 (1.78–2.81)	<0.001^a^
Employment Status				
Unemployment	ref		ref	
Employment	1.02 (0.85–1.21)	0.861	1.55 (1.26–1.91)	<0.001^a^
Wealth index				
Poorest	ref		ref	
Poorer	1.66 (1.27–2.16)	<0.001^a^	1.59 (1.16–2.17)	0.004^a^
Middle	1.93 (1.50–2.48)	<0.001^a^	1.67 (1.21–2.3)	0.002^a^
Richer	1.89 (1.47–2.44)	<0.001^a^	1.46 (1.05–2.04)	0.026^a^
Richest	2.88 (2.19–3.79)	<0.001^a^	2.73 (1.84–4.05)	<0.001^a^
Media exposure				
No access	ref		ref	
Access to at least one media	1.61 (1.35–1.93)	<0.001^a^	1.24 (1.01–1.53)	0.043^a^
Currently using tobacco products				
No	ref		ref	
Yes	0.61 (0.48–0.78)	<0.001^a^	0.54 (0.37–0.77)	0.001^a^
Awareness of other STIs				
No	ref		ref	
Yes	0.81 (0.67–0.99)	0.035^a^	0.78 (0.62–0.97)	0.028^a^
Level of knowledge of HIV/ AIDS				
Low (0–6)	ref		ref	
Moderate (7–8)	0.89 (0.72–1.09)	0.254	0.75 (0.59–0.95)	0.016^a^
High (9–11)	0.93 (0.78–1.12)	0.464	0.73 (0.57–0.94)	0.013^a^
Level of knowledge of contraception				
Low (0–1)	ref		ref	
Moderate (2)	1.18 (0.98–1.43)	0.082	1.06 (0.84–1.35)	0.615
High (3–4)	1.19 (0.96–1.48)	0.107	1.24 (0.96–1.6)	0.099

^a^ Significant at *p*-value < 0.05,.

## Discussion

This study examined sexual abstinence and its associated factors among Myanmar's youth aged 15–24, using data from the Myanmar Demographic and Health Survey (MDHS) 2015–16. The findings revealed a 71.2% prevalence of sexual abstinence among young people, with several sociodemographic and knowledge-related variables strongly associated with abstinence. These findings are critical for informing public policy and designing targeted sexual and reproductive health (SRH) interventions for Myanmar's youth.

We discovered that approximately 71% of Myanmar's youth aged 15–24 reported sexual abstinence. The abstinence rate here is higher than in other countries. A study from Côte d'Ivoire, for example, reported a primary abstinence prevalence rate of 33% (17), while another from North West Province, South Africa, reported a rate of 68% (5). The higher prevalence in Myanmar could be attributed to a number of factors, including strong sociocultural norms and religious beliefs that encourage abstinence until marriage, limited SRH education and services for youth, and a restrictive legal and policy environment surrounding youth sexual activity. This shows that widespread sexual abstinence remains a prominent behavioral norm among Myanmar's adolescents. This could be worsened by social expectations, family pressure, and restricted access to sexual health information and treatments, particularly among young women in rural communities. Nonetheless, nearly one-third of youth engage in sexual activity, highlighting the significance of risk reduction and comprehensive sexuality education. Older youths (20–24 years) were significantly less likely to practice abstinence than those aged 15–19 years. This age-related decline in abstinence is consistent with data from Nepal, Sub-Saharan African countries, and Côte d'Ivoire (1, 6, 17). Programs should prioritize sexual abstinence and SRH education in early adolescence, since risk rises with age. Female youths were less likely than males to indicate abstinence, which differed from trends reported in several African regions (6, 27, 28). This is because in Myanmar, females are more likely than males to marry at younger ages, and marriage is closely linked to sexual initiation (29). Thus, lesser abstinence among females may be due to earlier marriage rather than increased premarital sexual activity. When addressing abstinence and sexual behavior among Myanmar youths, sexual and reproductive health programs should consider gendered marital patterns as well as cultural expectations. Young people with a higher education level were more than twice as likely to abstain as those with a lower education level. Comparable research conducted throughout Africa confirm that increasing education is an important protective factor for abstinence (17, 30). This supports investment in girls' and boys' education as a structural driver of adolescent sexual and reproductive health and risk reduction. Employment was also positively associated with abstinence. This finding contradicts a study in Nigeria, which found that working youth were less likely to refrain from sexual activity (31). In the current study, this link appears to be consistent with broader socioeconomic patterns, since higher household wealth was independently associated with abstinence. Similar relationships between poorer socioeconomic status and lesser abstinence have been seen in other African settings (32). Together, these data imply that structural socioeconomic conditions, rather than work contexts, may play an essential role in determining youth sexual behavior. These findings highlight the need for sexual and reproductive health (SRH) interventions that address socioeconomic disparities, with a focus on youth from economically disadvantaged families. Furthermore, youths who were exposed to at least one type of media on a weekly basis were more likely to abstain. The DHS measures media exposure as access to newspapers, radio, or television, but it does not account for the nature or substance of the information accessed. Media exposure has been demonstrated to raise awareness and encourage preventative health behaviors such as abstinence and safe sex (33). Positive, informative media exposure, when carefully crafted, has the potential to reinforce abstinence and delay sexual debut (34, 35). Current tobacco users were significantly less likely to abstain. Other studies have consistently linked substance use, including tobacco use, to increased sexual risk-taking and lower abstinence (36, 37). Integrated approaches to multiple risk behaviors (tobacco, alcohol, and risky sex) should be addressed in SRH education.

Youth who reported STI awareness were less likely to abstain from sexual activity. In contrast to other study that reported a positive association (38), the present study showed a different pattern. Because this measure captured awareness rather than comprehensive knowledge, this connection most likely reflected increased information exposure among adolescents who are sexually active rather than a causal influence of STI knowledge on sexual behavior. Similarly, higher HIV/AIDS knowledge was associated with lower odds of sexual abstinence. Although prior studies suggest that greater HIV/ AIDS knowledge is associated with safer sexual behaviors (17, 39), the observed association in this study may be due to reverse causation rather than a protective impact of knowledge. The direction of this link cannot be determined based on the cross-sectional design utilized in the original study. Sexually active youths may be more likely to seek, receive, or retain information about HIV/AIDS and STIs following sexual debut, through health services, education programs, or personal experience. This underscores the need for longitudinal research to clarify causal pathways and highlights the importance of delivering comprehensive sexual and reproductive health education before sexual debut, while continuing to support risk-reduction among sexually active youths.

However, recent studies suggest that youth sexual behaviors, including sexual abstinence, have been substantially influenced by the COVID-19 pandemic and rapid technological advancements. Pandemic-related school closures, mobility restrictions, and reduced peer interaction were associated with delayed sexual debut and increased abstinence among adolescents in several settings, while also limiting access to sexual and reproductive health information and services for those who were sexually active (40, 41). Concurrently, increased use of digital technologies and social media has been associated with greater exposure to sexual content and online interactions, which may contribute to earlier sexual initiation and decreased abstinence for youths (42, 43). These contrasting trends highlight the need for future studies in Myanmar to examine how post-COVID-19 conditions and rapid technological advancements have reshaped youth sexual behaviors, including changes in sexual abstinence, sexual debut, and digitally mediated sexual interactions.

### Limitations and strengths

The study has several limitations. First, because the data was gathered in a cross-sectional study, it is impossible to draw causal associations between the identified factors and sexual abstinence behavior. Second, media exposure was measured as a composite indicator of access to newspapers, radio, or television, but it did not include media content, message quality, or digital media use. Third, STI knowledge was tested using a single proxy indicator, which does not account for STI-related depth, accuracy, or misconceptions, and should be considered when interpreting the data. Furthermore, relationships reported between sexual abstinence, HIV/AIDS knowledge, and STI awareness may indicate reverse causation, as information acquisition could occur after sexual debut. Finally, the analysis was based on self-reported data, which may be subject to recall and social desirability bias, especially given the sensitive nature of sexual behavior in Myanmar's cultural context. The dataset also lacked information on religion, lifestyle factors like alcohol drinking, relationship dynamics, and peer and parental influences, which may have resulted in residual confounding.

Furthermore, the data used in this study were collected in 2015–2016, nearly a decade before the present analysis. Since that time, Myanmar has experienced substantial social, political, and technological changes, including a period of democratic transition, the COVID-19 pandemic, and the rapid expansion of internet and smartphone access among youths before the political changes in 2021. These shifts have altered patterns of social interaction, access to information, and exposure to digital media, all of which may influence youth sexual behaviors, including sexual abstinence. Such contextual changes are not captured in the MDHS 2015–2016 data and may limit the applicability of the findings to current and post-pandemic contexts, highlighting the need for updated nationally representative data.

Nonetheless, the study's strengths include the use of a large, nationally representative sample with appropriate weighting and strong statistical analyses. Unlike previous studies that focused on individual regions or youth groups, this study provides the first comprehensive picture of youth sexual abstinence and its correlates in Myanmar, providing critical evidence for policy and programming.

### Policy and practice implications

The findings highlight the need to tailor sexual and reproductive health (SRH) strategies in Myanmar to address the specific socioeconomic and behavioral factors associated with sexual abstinence among youths. Given the significant associations observed with education level, employment status, household wealth, mass media exposure, and tobacco use, SRH policies should extend beyond abstinence-only messaging to incorporate structural and behavioral interventions.

In particular, policies that promote continued school participation and reduce school dropout, expand youth employment and livelihood opportunities, and address socioeconomic inequalities should be implemented to support delayed sexual initiation among youths. Given the positive association between media exposure and abstinence, youth-focused mass media strategies should be strengthened to reinforce preventive SRH messages. In addition, the lower likelihood of abstinence among tobacco users indicates the need to integrate substance-use prevention into SRH programming to promote healthier sexual decision-making among Myanmar youths.

## Conclusion

This study provides nationally representative evidence on the prevalence of sexual abstinence and its associated factors among youths aged 15–24 in Myanmar. Sexual abstinence was significantly associated with age, education level, sex, employment status, household wealth, media exposure, and tobacco use, highlighting the importance of socioeconomic and behavioral contexts in shaping youth sexual behavior.

These findings underscore the need for interventions that support school retention, address socioeconomic disadvantage, promote positive media engagement, and integrate substance-use prevention within sexual and reproductive health initiatives aimed at youths. Given the cross-sectional design and the age of the data, future longitudinal and updated nationally representative studies are needed to better understand changing patterns of sexual abstinence and their determinants among Myanmar youths.

## Data Availability

The raw data supporting the conclusions of this article will be made available by the authors, without undue reservation.
